# Antiviral antibody responses to systemic administration of an oncolytic RNA virus: the impact of standard concomitant anticancer chemotherapies

**DOI:** 10.1136/jitc-2021-002673

**Published:** 2021-07-21

**Authors:** Victoria Roulstone, David Mansfield, Robert J Harris, Katie Twigger, Christine White, Johann de Bono, James Spicer, Sophia N Karagiannis, Richard Vile, Hardev Pandha, Alan Melcher, Kevin Harrington

**Affiliations:** 1 Radiotherapy and Imaging, The Institute of Cancer Research, London, UK; 2 St John's Institute of Dermatology, Guy's Hospital, London, UK; 3 Department of Molecular Medicine, Mayo Clinic, Rochester, Minnesota, USA; 4 Faculty of Health and Medical Sciences, University of Surrey, Guildford, UK

**Keywords:** immunity, humoral, immunomodulation, oncolytic virotherapy, oncolytic viruses

## Abstract

**Background:**

Oncolytic reovirus therapy for cancer induces a typical antiviral response to this RNA virus, including neutralizing antibodies. Concomitant treatment with cytotoxic chemotherapies has been hypothesized to improve the therapeutic potential of the virus. Chemotherapy side effects can include immunosuppression, which may slow the rate of the antiviral antibody response, as well as potentially make the patient more vulnerable to viral infection.

**Method:**

Reovirus neutralizing antibody data were aggregated from separate phase I clinical trials of reovirus administered as a single agent or in combination with gemcitabine, docetaxel, carboplatin and paclitaxel doublet or cyclophosphamide. In addition, the kinetics of individual antibody isotypes were profiled in sera collected in these trials.

**Results:**

These data demonstrate preserved antiviral antibody responses, with only moderately reduced kinetics with some drugs, most notably gemcitabine. All patients ultimately produced an effective neutralizing antibody response.

**Conclusion:**

Patients’ responses to infection by reovirus are largely unaffected by the concomitant drug treatments tested, providing confidence that RNA viral treatment or infection is compatible with standard of care treatments.

## Introduction

Mammalian orthoreovirus type 3 Dearing (hereafter referred to as reovirus) is a wild-type double-stranded RNA virus. While reovirus is non-pathogenic in humans, it has been shown to replicate selectively in cells that have an activated or mutated Ras signaling pathway^1^ and this inspired research into its use as an oncolytic virus. Later research indicated that the oncolytic activity of reovirus may be contingent on a more complex and nuanced mechanism than simply being Ras enabled.^2 3^

It has been suggested that antireoviral antibody responses may hinder the antitumor efficacy of reovirus. It has also been postulated that frequent, high doses of virus given before an antibody response peaks or cytotoxic chemotherapy-mediated attenuation of the antibody titer might enhance systemic delivery of virus to tumor tissue.^4^ More recently, preclinical data have suggested that the antireovirus neutralizing antibody response may, paradoxically, enhance therapy in mouse models.^5 6^ This effect, which occurs after cotreatment with granulocyte-macrophage colony-stimulating factor, was predicated on enhanced monocyte/macrophage virus carriage and delivery to tumor. It is not known if this phenomenon occurs in patients.

Reovirus has been tested as a single agent in multiple clinical trials in patients with advanced cancers, initially intratumorally^7^ and then intravenously.^8 9^ No dose-limiting toxicities were observed, and clear signs of single-agent activity were observed, with a 37% response rate in the first trial of intratumoural administration^10^ and a 45% response rate in the first trial of intravenous administration, improving to 67% in patients with confirmed viral shedding.^11^ Subsequently, preclinical and clinical studies combining reovirus with standard anticancer chemotherapies were conducted in an attempt to further improve responses.^12–17^ These phase I trials, which variously involved combinations with gemcitabine, platinum and taxanes, primarily looked for safety, tolerability and signals of efficacy, but data were also collected on the effect of chemotherapy on antiviral humoral immune responses. In particular, studies on cyclophosphamide were conducted with the premeditated intention of blunting antiviral antibody responses to allow more effective systemic delivery. Promising preclinical data^18–20^ underpinned that phase I trial of escalating doses of cyclophosphamide with a primary endpoint of modulating neutralizing antireoviral antibody (NARA) responses. The combination was safe but, even at myelotoxic doses of cyclophosphamide, neither consistent modulation of NARA responses nor enhanced antitumor efficacy was achieved.^21^

While these studies were designed to enhance the efficacy of reovirus, they also provide important insights into the ability of patients with cancer to respond to and clear viral infection despite receiving systemic immunosuppressive therapies, an important consideration for combination viral therapies.

Here, in what is to the best of our knowledge a unique dataset, we compared NARA responses in patients treated in phase I/II studies of intravenous reovirus either as a single agent or in combination with gemcitabine, docetaxel, carboplatin and paclitaxel doublet or cyclophosphamide.

## Materials and methods

### Cell lines

L929 (mouse fibroblast; Oncolytics Biotech) were cultured in DMEM. Media was supplemented with 5% (v/v) Fetal Calf Serum (FCS) (or 2% (v/v) FCS for plating media), 1% (v/v) glutamine, and 0.5% (v/v) penicillin/streptomycin.

### Reovirus stocks

Mammalian orthoreovirus type 3 Dearing (Pelareorep) in PBS was obtained from Oncolytics Biotech (Calgary, AB, Canada) and stored at −80°C. Viral titer was regularly confirmed by TCID_50_ assay.

### Antibody analysis

Samples were collected from patients at various time points within their treatment course according to the protocol-defined schedules. Clotted blood samples were taken and within 4 hours, centrifuged at 3000 rpm for 10 min at room temperature and stored at −80°C. Patient sera were heat inactivated at 56°C for 30 min prior to antibody analysis. To calculate patients’ antibody titers, a modified neutralizing antibody assay was employed as described previously.^4^ Briefly, a known titer of reovirus was incubated with serial dilutions of patients’ serum and the neutralization of virus particles was assessed by loss of ability to kill a monolayer of L929 cells (analyzed by the MTT assay). A goat polyclonal antibody was used as a positive control.

### Calculation of antibody titer

For accuracy, two methods of calculating antibody titers were employed. The highest dilution that displayed neutralization (>80% cell killing) in any of the replicate wells was classed as the endpoint titer. The second method of calculating antibody titer used the highest dilution of patient serum that gave >50% neutralization in the replicate wells (eg, <80% cell killing in over half of replicate wells); this is classed as the halfpoint titer.

### Serum antibody isotyping time-course in patients receiving reoviral intravenous injections

To quantify changes in patient serum antibody isotype titers in response to reoviral administration, high-throughput 7-plex antibody isotyping magnetic bead-based Luminex assays (Thermo Fisher) were performed according to manufacturer’s instructions. Serum concentrations of IgG1, IgG2, IgG3, IgG4, IgM, IgA and IgE were quantified at defined time-points: pretreatment (0), 5, 8 and 15 days following the initiation of each dosage cycle. Serum was stored at −80°C and thawed on ice prior to analysis.

### Clinical trials

Antibody data were collected from various phase I/II clinical trials administering RT3D intravenously as a single agent,^8^ in combination with gemcitabine,^16^ docetaxel,^17^ in combination with the two chemotherapeutic agents paclitaxel and carboplatin,^15^ and in combination with cyclophosphamide,^21^ in patients with advanced malignancies.

### Statistics

Low sample number for some cohorts at individual time points reduced the power of statistical comparisons at those time points. To overcome this, where noted, the area under the curve from baseline to C2D1 was calculated for each cohort to create comparisons of NARA response over the first 2 weeks of treatment that can be subjected to statistical analysis. Kruskal-Wallis non-parametric tests, and two-sided paired t-tests were used to compare between and within cohorts, respectively. Data are presented as mean±SEM, and p values were reported as: ns=not significant, p<0.05 (*) and p<0.01 (**). All statistical analysis was performed using GraphPad Prism V.7.0.

## Results

### Patients

**Figure 1:**
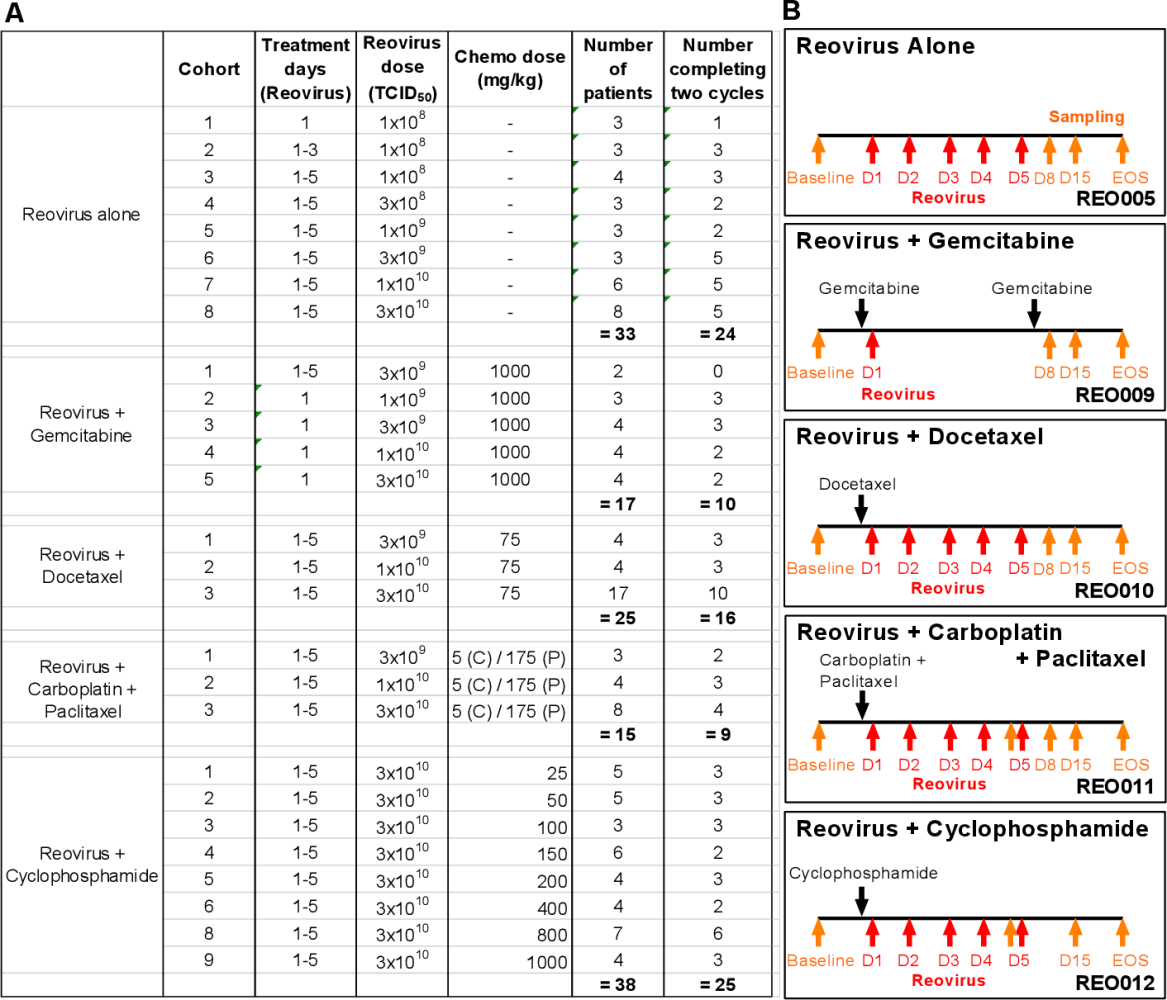
Doses and treatment schedules for phase I studies of reovirus and chemotherapy. (A) Clinical trials include reovirus (REO) administered intravenously as a single-agent (REO005) or in combination with chemotherapy: gemcitabine (REO009), docetaxel (REO010), carboplatin and paclitaxel (REO011), and cyclophosphamide (REO012). With the exception of REO009, reovirus was given consecutively for the first 5 days of the treatment cycle. For REO005 and REO012, each cycle lasted 28 days, and for REO009, REO010 and REO011, each cycle lasted 21 days. (B) Blood samples for antibody analysis were collected as indicated by the orange arrows. Reovirus was administered within the first 5 days (days 1–5) of the treatment cycle as indicated by the red arrows. EOS, end of study.

Details of patients who were enrolled in a series of clinical trials of reovirus, with or without different chemotherapies, and the relevant treatment and sampling schedules are summarized in figure 1. The different combinations allowed us to address the effect of multiple chemotherapy drugs on the NARA response in patients.

### Neutralizing antireovirus antibody titers

**Figure 2:**
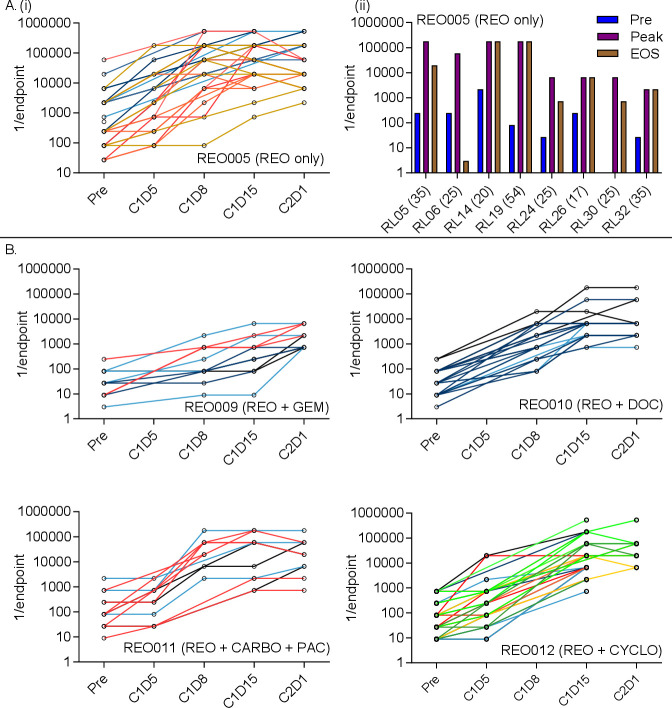
NARA titers rise in response to reovirus infusion in patients with cancer. (A) (i) Patients received intravenous reovirus (IV) as a single agent (REO005). Individual patients’ NARA titers are shown throughout the first two cycles of treatment, with each line representing an individual patient (left) (C=cycle, D=day). (ii) For patients where an end of study (EOS) sample was taken, the titer was plotted against the overall highest antibody titer (peak) and pretreatment titer (pre). Bracketed numbers show the elapsed time (days) between the last infusion of reovirus and EOS sampling. B. Four additional trials tested reovirus in combination with gemcitabine (REO009), docetaxel (REO010), carboplatin and paclitaxel (REO011) or cyclophosphamide (REO012). Individual patients’ titers were plotted over the first two courses of treatment. Data are shown using the endpoint method to calculate titers.

Antibody levels against reovirus were assessed by a modified neutralizing antibody assay as described previously.^4^ Individual patients’ NARA responses to intravenous reovirus monotherapy (REO005 trial) are shown in figure 2A(i). Despite heat inactivation of complement, all patients’ serum displayed a limited pre-existing ability to neutralize reovirus prior to reovirus treatment, although this baseline was variable, consistent with the known exposure to reovirus of the general population.^22 23^ NARA titers generally began to increase 5–8 days after the first reovirus infusion. Patients most often reached peak titer around 15 days postinfusion.

For patients who completed at least two cycles of treatment and where an end-of-study sample was available, NARA endpoint titers pretreatment, at peak and at the end-of-study are summarized in figure 2A(i) and show that peak titers are maintained at or near the peak until end of study for most patients, up to 54 days following the last infusion. Complete endpoint antibody titers at all measured time points in REO005 are summarized in online supplemental figure 1A–H.

10.1136/jitc-2021-002673.supp1 Supplementary data

Individual patients’ NARA responses, when reovirus was given in combination with gemcitabine (REO009), docetaxel (REO010), carboplatin plus paclitaxel (REO011) and cyclophosphamide (REO012) are shown in figure 2B. Patients’ NARA responses appear similar to those seen with reovirus monotherapy, peak titers being reached around day 15; however, the peak titers themselves were suppressed with gemcitabine treatment, being 1–2 logs lower than in the other trials. This apparent difference may be due to lower total doses of reovirus (one dose vs five consecutive doses), rather than due to concomitant gemcitabine treatment.

### NARA responses in patients treated with or without combination chemotherapy

**Figure 3:**
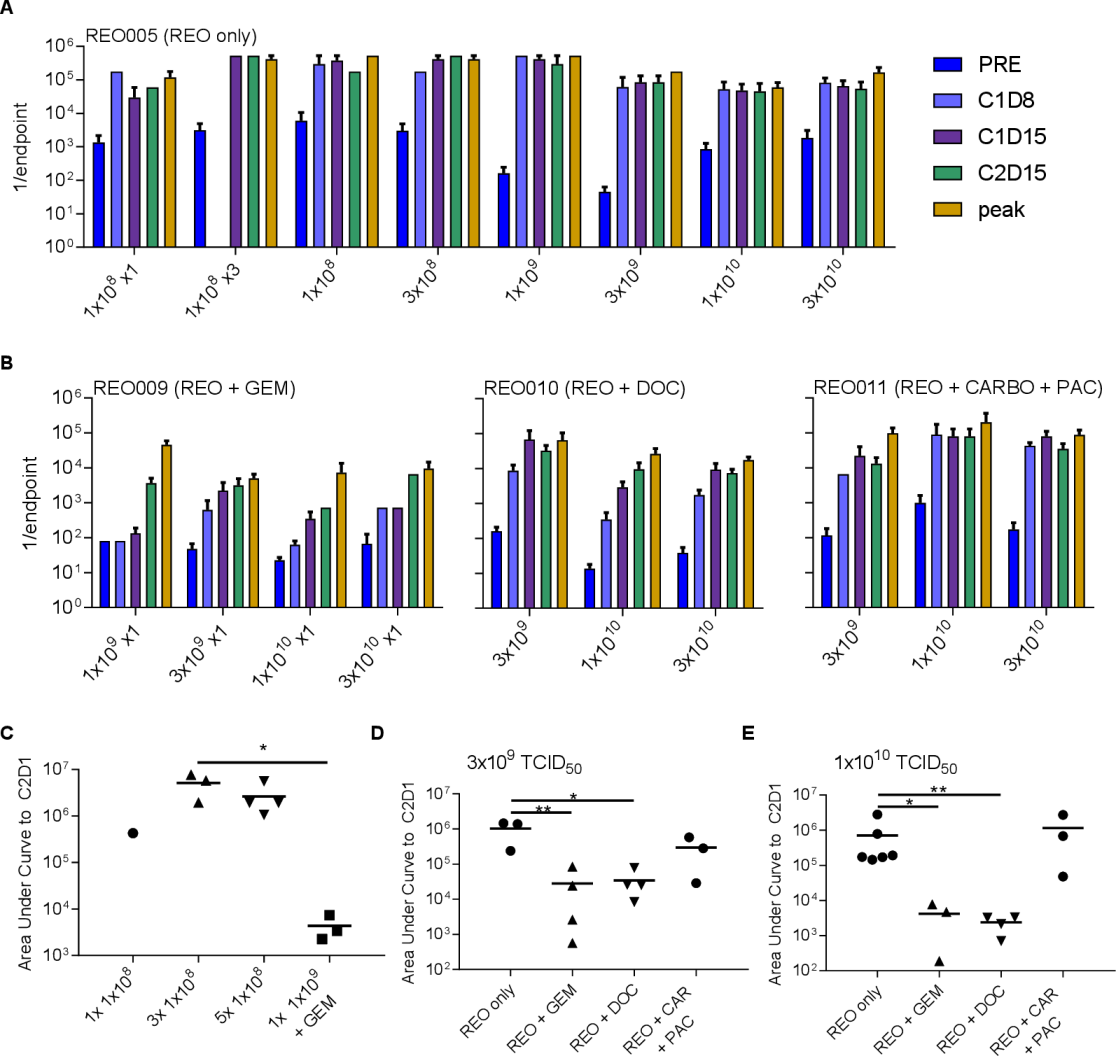
Effects of virus dose and chemotherapy combination on NARA kinetics. (A) Reovirus dose and NARA response for patients treated with reovirus alone (REO005). Patients are grouped by cohort, each with increasing doses of reovirus. Reovirus infusions were given five times, once each day over 5 days (unless shown otherwise). (B) Reovirus dose and NARA response grouped by incremental doses of reovirus in combination with fixed doses of gemcitabine, docetaxel or carboplatin/paclitaxel doublet. For REO009, only one dose of reovirus was given per cycle due to toxicity reasons, and REO010 and REO011 represent five doses per cycle. (C) Comparison of NARA responses after 1, 3 or 5 doses of reovirus at 1×10^8^ TCID_50_ and a single dose of 1×10^9^ TCID_50_ given with gemcitabine. (D) Comparison of NARA titer kinetics with/without gemcitabine, docetaxel or carboplatin and paclitaxel at 3×10^9^ TCID_50_ and (E) at 1×10^10^ TCID_50_. Error bars=SEM. NARA, neutralizing antireoviral antibody.

For patients treated with reovirus alone (REO005), average NARA titers are shown per cohort in figure 3A. Most patients receiving reovirus alone (REO005) appeared to develop a marked antibody response by C1D8 and peak levels of antireovirus antibody levels within their first cycle of treatment by days 8–15. There was no significant increase in peak NARA titers associated with increasing reovirus dose.

Figure 3B shows the same information for patients receiving reovirus plus chemotherapy. Again, no significant increases in peak NARA titers were observed in response to increasing reovirus doses for any drug combination. NARA responses appear to be delayed in combinations with docetaxel or gemcitabine (but less so with carboplatin/paclitaxel), as illustrated by the longer time taken for titers to approach peak levels (compare with data in figure 3A). This suggests that there may be a longer window at the start of certain combination studies in which to ‘load’ virus before antiviral antibody levels rise too high.

It is important to note that a reduced reovirus dose frequency was used for the patients receiving gemcitabine because of observed hepatic and cardiac toxicities within the first two patients (excluded from all analyses here because of incomplete acquisition of data). It is also important to note that viral replication was not implicated directly in these toxicities and the virus remained non-pathogenic. However, reovirus dosing was reduced from the standard five doses (days 1–5) per cycle used in the other trials to one dose (on day 1), and treatment-related toxicities were not seen with this regimen. It is possible that this dose reduction was responsible for the lower NARA titers, and to address this further, the ‘area under the curve’ (AUC) of antibody levels, from baseline to C2D1, was calculated for each patient in cohorts 1, 2 and 3 of the single agent REO005 trial who received a dose of 1×10^8^ TCID_50_ once, thrice or on five consecutive days, respectively. This escalating frequency of reovirus dosing had no significant effect on the AUC NARA response in the early cohorts of REO005, although only one data point was available for the single infusion cohort, as dictated by the trial design (figure 3C). Additionally, the higher 1×10^9^ TCID_50_ dose used with gemcitabine in cohort 2 of REO009 shows extensive suppression of the NARA response, with an area under the curve several logs lower than all the reovirus alone cohorts, despite the higher total virus dose. In addition, the titer remains close to the baseline level until day 15 (online supplemental figure 3A). This analysis shows that the suppression of the NARA response observed with gemcitabine was likely a genuine drug effect, rather than a dose-frequency effect. Importantly, after the number of doses of reovirus in this regimen was reduced from 5 to 1 (and all data presented here reflect this dose reduction), the combination of reovirus and gemcitabine was well tolerated.

10.1136/jitc-2021-002673.supp5 Supplementary data

Because REO005, REO010 and REO011 used similar doses and treatment regimens, it was possible directly to compare NARA responses to equivalent viral doses in the presence or absence of chemotherapy. Cohort 1 in REO010 and in REO011 and cohort 6 in REO005 all received reovirus at the same dose of 3×10^9^ TCID_50_, on five consecutive days; cohort 3 of REO009 received a single dose of 3×10^9^ TCID_50_. This allowed a direct comparison of the effects on NARA titers of docetaxel, carboplatin/paclitaxel doublet and a close comparison for gemcitabine. Likewise, cohort 5 from REO009 and cohort 2 from REO010 and from REO011 can be compared with cohort 7 in REO005 to make the same comparison for patients receiving 1×10^10^ TCID_50_ doses (refer to figure 1A).

Again, area under the curve to C2D1 was calculated for these comparisons, showing that at the 3×10^9^ dose (figure 3D), the NARA titer was significantly suppressed by gemcitabine and docetaxel but not by carboplatin/paclitaxel. Although docetaxel was suppressive, this effect was short lived, and the peak titer was still reached by C1D15, at a comparable level to titers for patients receiving reovirus alone. In contrast, gemcitabine suppression lasts longer and reduced the final peak titer approximately 10-fold (online supplemental figure 3B). At the 1×10^10^ dose, significant suppressive effects were seen again (figure 3E) and for longer duration, with suppression in the titer at C1D15 still apparent for both gemcitabine and docetaxel (online supplemental figure 3C). Again, no suppressive effect of carboplatin/paclitaxel was seen.

### Cyclophosphamide given to induce immunosuppression has modest effect on NARA responses

**Figure 4:**
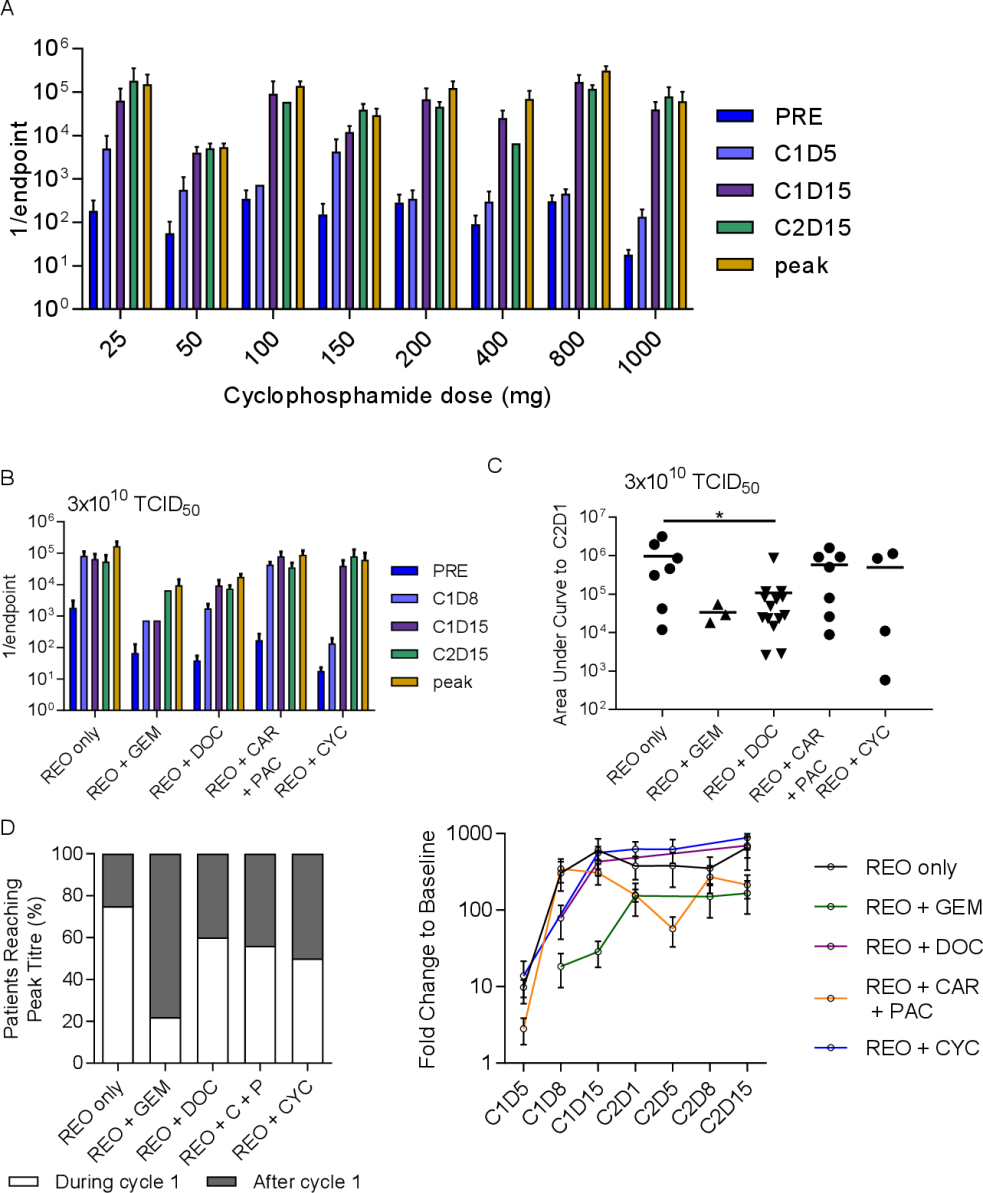
Cyclophosphamide treatment to abrogate the immune response was not more effective than other treatments. (A) Summarized antibody titers for each cohort in the REO0012 trial where 3×10^10^ TCID_50_ was given for 5 days at the start of each cycle alongside cyclophosphamide at escalating doses in each successive cohort. (B) A comparison of the NARA tiers for patients receiving the same dose of virus alongside different chemotherapy agents or none in each of the other trials. (C) Area under the curve analysis up to C2D1 for the same patients. *P<0.05 D analysis of all patients in each trial (regardless of dose levels), grouping patients whose titers peaked in cycle 1 or at a later time point. (E) Average NARA titers for all patients on each trial over the course of treatment. Error bars=SEM. NARA, neutralizing antireoviral antibody.

The immunosuppressive drug cyclophosphamide was given in increasing doses in combination with a fixed dose of reovirus (3×10^10^ TCID_50_) in the trial REO012, specifically to find a dose that would delay the NARA response, theoretically allowing enhanced tumor colonization and replication of the virus. The NARA response to 3×10^10^ TCID_50_ concomitantly with increasing doses of cyclophosphamide is shown in figure 4A. No clear cyclophosphamide dose effect was apparent in these NARA titers. As previously, we compared these data with cohorts receiving similar reovirus doses in the other trials, that is, cohort 8 from REO005, cohort 5 from REO009 and cohort 3 from both REO010 and REO011, compared with the cohort with the highest dose of cyclophosphamide (1000 mg), cohort 9 from REO012. Compared with the other chemotherapy agents at this dose of reovirus, cyclophosphamide appears to slow the initial NARA response, with titers lower at C1D8 than for all comparators (figure 4B). Analysis of area under the curve to C2D1 shows that half of the patients appear to have had suppressed NARA responses, but there was no overall statistical significance (figure 4C). Notably, the suppression seen previously for gemcitabine and docetaxel remains clear at this higher dose of virus.

Given that the effect of escalating virus dose on NARA responses in each trial was minimal, it was possible to pool all the patients in each trial together to get a comprehensive view of how NARA titers were affected by the drug combinations. We assessed whether all patients on each trial, regardless of virus dose, reached peak titer during or after the first cycle of treatment (figure 4D). This showed that the most effective drug for delaying the peak NARA titer is gemcitabine, where 80% of patients reached peak NARA response in the later cycle. Cyclophosphamide was the second most effective but resulted in a later peak titer for only half of patients. To normalize for the potential effect of varying baseline titers, NARA titer fold change over time was analyzed for all patients in each trial (figure 4E). This shows that docetaxel and cyclophosphamide have a similar suppressive profile at C1D8 but that this was lost by C1D15, from which point the levels were similar to reovirus alone. Carboplatin/paclitaxel was the only combination that shows a dip in the antibody titer before the second cycle, and gemcitabine displays the slowest and overall lowest increase in titers.

### The NARA response was primarily IgG1; all isotypes were suppressed by docetaxel and gemcitabine

**Figure 5:**
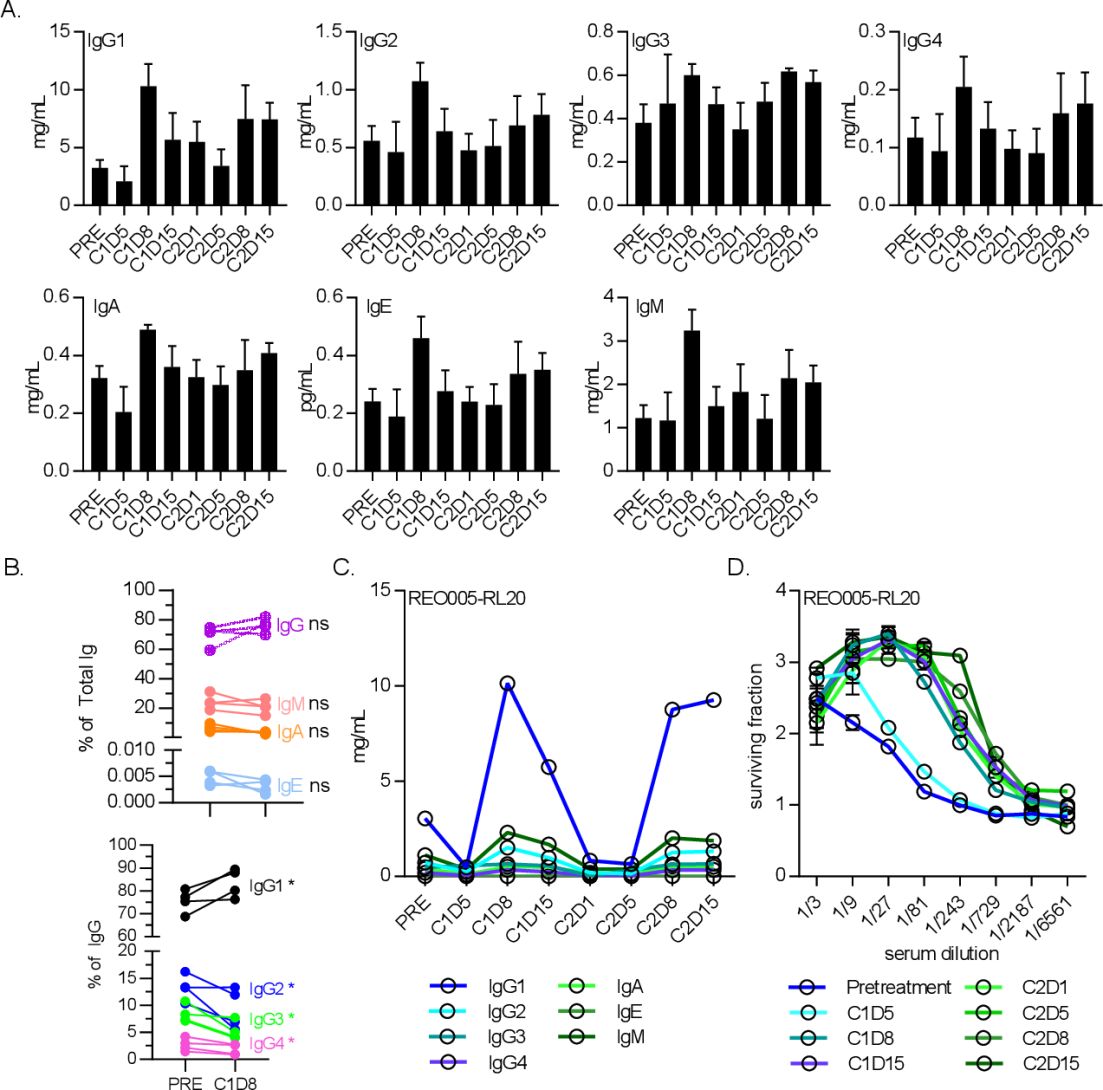
The antibody isotype IgG1 was the dominant responder to reovirus infection. (A) Levels of antibody isotypes IgG1, IgG2, IgG3 and IgG4, IgM, IgA and IgE for five patients receiving 1×10^10^ TCID_50_ doses of reovirus alone for 5 days at the start of each cycle. Error bars=SEM. (B) Antibody class profiles (top) and specific IgG isotype bias (bottom) between baseline and cycle 1 day 8 (C1D8). *P<0.05. (C) Antibody isotype levels throughout treatment of a representative patient, showing IgG1 as the main constituent increased at C1D8. (D) Neutralizing antireovirus antibody levels for the same patient.

To explore the NARA response in more detail, the total titers of antibody subtypes in blood sera were quantified to determine their individual kinetics in response to reovirus (figure 5). This quantification included all antibodies in circulation, not just reovirus-specific antibodies, but it was assumed that responses seen in the window of treatment were primarily antireovirus. Patients receiving 1×10^10^ TCID_50_ were assessed for IgG1, IgG2, IgG3, IgG4, IgM, IgA and IgE in blood serum. The kinetics of each isotype were broadly similar, all initially peaking at day 8 postinfusion before reverting to pretreatment levels before the second infusion (figure 5A). A response was seen in all isotypes, but the highest mean titer (across n=5 patients for each timepoint) was for IgG1 (10.3 mg/mL at C1D8), followed by IgM (3.2 mg/mL at C1D8), and the remaining isotypes approximately 1 log lower. Despite all isotype titers increasing in response to virus treatment, the IgG1 response was so overwhelmingly large that the percentage proportions of other isotypes were depressed in comparison (figure 5B). As a representative example, the kinetics of individual isotypes from a single patient were compared (figure 5C), clearly showing the overwhelming IgG1 response. Despite the total antibody titers returning to pretreatment levels between cycles of treatment, the NARA levels remained at peak titer from day 8 onwards (figure 5D). This indicates that the vast majority of antibodies produced, even if reovirus-specific, were not able to neutralize the virus, as the NARA titers do not decrease at all even when the total antibody titers return to pretreatment levels.

**Figure 6:**
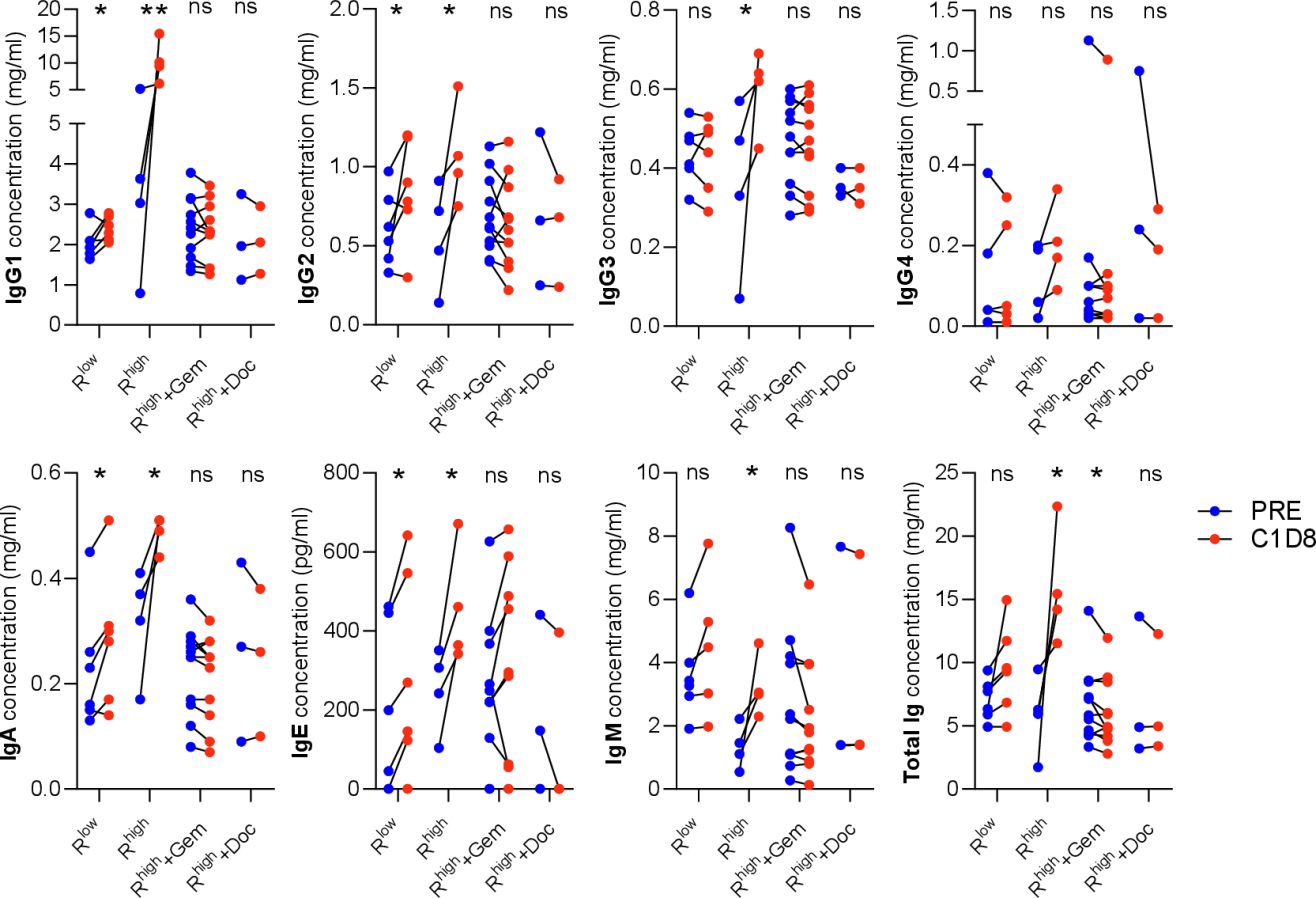
Effect of reovirus dose and chemotherapy agents on individual isotype concentrations. IgG1-4, IgA, IgE and IgM concentrations measured before treatment (PRE) and on C1D8. Low and high doses of reovirus and the high dose in combination with gemcitabine (Gem) and docetaxel (Doc) (R^low^ =<1×10^9^ TCID_50_, R^high^=1×10^10^ TCID_50_). *P<0.05; **p<0.01. ns, not significant.

The NARA data showed different levels and duration of NARA suppression by gemcitabine and docetaxel. To determine if these differences were due to isotype switching from IgG1 to a less effective isotype such as IgG4, sera from patients receiving the same 1×10^10^ TCID_50_ dose in combination with these drugs were also analyzed (figure 6).

The significant concentration increases seen in every isotype (except IgG4) between baseline and C1D8 were completely suppressed by both gemcitabine and docetaxel, with levels unchanged compared with baseline in each case. Total immunoglobulin concentration was significantly reduced by gemcitabine, but no significant change was seen with docetaxel. This observation is consistent with gemcitabine having a larger effect on the NARA responses observed.

The 1×10^10^ TCID_50_ dose was able to stimulate a broad range of isotypes, with all showing a significant increase in concentration except IgG4. Lower doses were also compared as the gemcitabine was given with just a single infusion of virus. These ranged from 1×10^8^ TCID_50_ given once, 1×10^8^ TCID_50_ given five times and 1×10^9^ TCID_50_ also given five times. These doses were grouped as R^low^ in figure 6 and appear to show a reduced IgG1 response compared with the higher dose (R^high^) as well as no significant IgG3 or IgM response, altogether resulting in no significant change in the total immunoglobulin concentration.

## Discussion

In these studies, we have been able to compare the immune responses of patients receiving intravenous reovirus with or without concomitant chemotherapy. Although the studies were performed at different times and samples analyzed over the course of several years, all the NARA data came from the same analytical laboratory using the same methodology.

All patients had low levels of pre-existing antibodies against reovirus before treatment, consistent with the ubiquitous nature of the virus, and all patients’ antibody titers increased further at some point after treatment. While most patients in these studies had undergone extensive therapy prior to trial entry, it appeared not to have affected their ability to raise antibodies against reovirus. Viral kinetic data gathered by PCR for reovirus genomes on peripheral blood serum in these trials was previously published.^8 16 17 21 24^ Across all the trials reovirus levels were below the detection threshold of the assay in the vast majority of samples tested, with numbers of patients testing positive being too low to draw any conclusions about the effects of antibody titers on rates of viral clearance.

Here, we saw that gemcitabine caused a significant attenuation of the NARA response. We also saw that a strategy specifically designed to attenuate the immune response using cyclophosphamide largely failed to achieve that goal. Doublet carboplatin and paclitaxel treatment had no initial effect on the antibody response, but the durability of response was reduced compared with other treatments and, ultimately, the peak titers reached were somewhat lower. Docetaxel treatment was very mildly suppressive in the first week but subsequent antibody titers were very similar to treatment with reovirus alone. It is worth noting the heterogeneity of the observed responses between patients within the same studies, demonstrating marked patient-to-patient variability.

Examining the complete antibody response during the course of treatment in more detail, we show that the response was primarily of the IgG1 isotype, which was the isotype primarily associated with response to viral proteins and the most potent inducer of complement-dependent cytotoxicity and antibody-dependent cellular cytotoxicity and antibody-dependent cellular phagocytosis. These processes are all involved in immune clearance of infected tumor cells that may cause subsequent recognition of tumour-specific antigens and activation of an antitumor response.^25 26^ We also saw that the circulating levels of IgG1 returned very quickly to baseline, without an associated reduction in NARA titer, indicating that a great many other clones were generated alongside the neutralizing clones. These may have consisted of non-neutralizing clones for virus antigens and clones targeting tumor antigens or self-antigens as a consequence of immune surveillance surrounding infected tumor cells.

The speed of the total antibody response, peaking rapidly in 8 days, suggests that these were mainly antibodies produced by reactivated memory B cells. If the response included antibodies against tumour-associated antigens, these were likely to be from memory B cells activated much earlier in the course of disease and subsequently suppressed. The total antibody response was also likely to include a number of self-antibodies. Autoreactive B cells have been shown to escape clonal deletion during maturation and later rapidly differentiate into plasma cells targeting self-antigens in response to pathogen-associated molecular patterns; however, these cells are short lived and self-antibodies rapidly return to low levels in normally regulated immune systems.^27^ With this large non-specific antibody response, it is possible that we lack the resolution to detect any virus-specific fluctuations. The observation that peak neutralizing titer was not reached until day 15 suggests that there is a disconnection between the total antibody response and the NARA response. Other studies have indicated by depletion experiments that reovirus-neutralizing antibodies in patient serum consist mainly of IgG and IgA, but not IgM.^5^

Due to the nature of the trials analyzed here, it is difficult to speculate on how the rate of antibody response affects clinical outcomes for patients. The trials were designed to test safety rather than efficacy and populated by patients with advanced stage cancers of many different types being treated palliatively. The history and prior treatments these patients had experienced also varied widely. It could be hypothesized that the prior treatments might have affected the ability to mount an antibody response. Although there was some variation in peak titer reached on an individual level, identifying the source of this variation with so many confounding factors would be difficult. Comparison with ‘normal’ serum in healthy volunteers is not possible, as these data do not exist.

The interactions between antibodies and the reovirus particle are more complex than a simple neutralization reaction. While neutralizing antibodies immediately inactivate the virion, non-neutralizing antibodies are not without function and can still result in complement activation and cell-based neutralization.^28^ As early as the 1960s, infectious antibody-reovirus complexes were hypothesized and were shown to be infectious in a later study that showed that the virion in the infectious complexes was not morphologically altered and, therefore, these complexes could be a common occurrence.^29^ More recently, it was shown in human in vitro studies that while antibody-neutralized reovirus could not directly infect tumor cells, the complex could be loaded onto monocytes, where it is internalized and restored to an infectious state. The virion then does not infect the monocyte but can be handed off to tumor cells by direct cell–cell contact, where viral replication does occur, ultimately causing oncolysis.^5^

This observation has been replicated in clinical trials, where reovirus was reported to associate beneficially with peripheral blood mononuclear cells (PBMCs) in such a way that it retained infectivity, circulated as a passenger and could be unloaded to infect target cells in vitro.^30^ This was also reported on patient PBMCs collected up to 2 weeks following reovirus infusion, in subsequent cycles of treatment and despite the development of high NARA titers.^21^ Therefore, it may be the case that transport in/on the PBMCs may be a mechanism to evade NARA, and immune cells may play a beneficial role in systemic viral delivery, potentially shielding reovirus for tumor targeting. In a number of trials, viral replication was detected in tumor biopsies even at time points by which a strong neutralizing antibody response had occurred,^8 9 30 31^ further indicating that the virus is able to persist despite the induction of neutralizing antibodies. Ex vivo expansion and loading of immune cells with non-neutralized reovirus prior to infusion is a therapeutic option that has also been explored and shown to be capable of delivery of virus to tumors.^32 33^

In the past 5 years, it has become widely accepted that that the immune system has an important and beneficial role in tumor control and can be harnessed to induce durable complete responses in patients using immunotherapies, but the role of the humoral response has been largely overlooked.^34^ It has also become increasingly clear that immune clearance of virally infected tumor cells can be a powerful stimulator of antitumor immunity.^35^ A more recent study using a reovirus and gemcitabine combination in pancreatic adenocarcinoma found evidence of viral replication in a tumor,^36^ noted a possible increase in tumor expression of the immune checkpoint marker programmed death-ligand 1 and suggested that additional anti-programmed cell death protein 1 therapy may be beneficial; this was further explored in a following study adding pembrolizumab to the combination with promising results.^31^ However, immunosuppression and treatment scheduling may be significant factors and must be considered carefully. The potential for reovirus to synergise with other immunotherapies is the subject of many additional studies,^37 38^ but it seems likely that a well-functioning immune system will be important in ensuring durable responses in this approach and immunosuppression is, therefore, to be avoided.

An understanding of the interaction between standard cancer treatments, the immune system and oncolytic viruses will help us understand, develop and improve future viral and immunotherapies for cancer. In this sense, these data provide an insight as to how the immune system responds to viral infection and how existing chemotherapy treatments can affect such responses. This information also has applications beyond the scope of oncolytic viruses, in the realm of infectious diseases.

10.1136/jitc-2021-002673.supp2 Supplementary data

10.1136/jitc-2021-002673.supp3 Supplementary data

10.1136/jitc-2021-002673.supp4 Supplementary data

## Data Availability

Data are available on reasonable request. Data are available on request from the corresponding author.
