# Correction: Antiviral antibody responses to systemic administration of an oncolytic RNA virus: the impact of standard concomitant anticancer chemotherapies

**DOI:** 10.1136/jitc-2021-002673corr1

**Published:** 2022-10-25

Roulstone V, Mansfield D, Harris RJ, *et al*. Antiviral antibody responses to systemic administration of an oncolytic RNA virus: the impact of standard concomitant anticancer chemotherapies. *J Immunother Cancer* 2021;9:e002673. doi: 10.1136/jitc-2021-002673

Victoria Roulstone and David Mansfield are now listed as joint first authors.

