# Supplementary figures and images for: Antiviral antibody responses to systemic administration of an oncolytic RNA virus: the impact of standard concomitant anticancer chemotherapies
Source: J Immunother Cancer. 2021 Jul 21;9(7):e002673. doi: 10.1136/jitc-2021-002673 (PMC8728387; doi:10.1136/jitc-2021-002673)

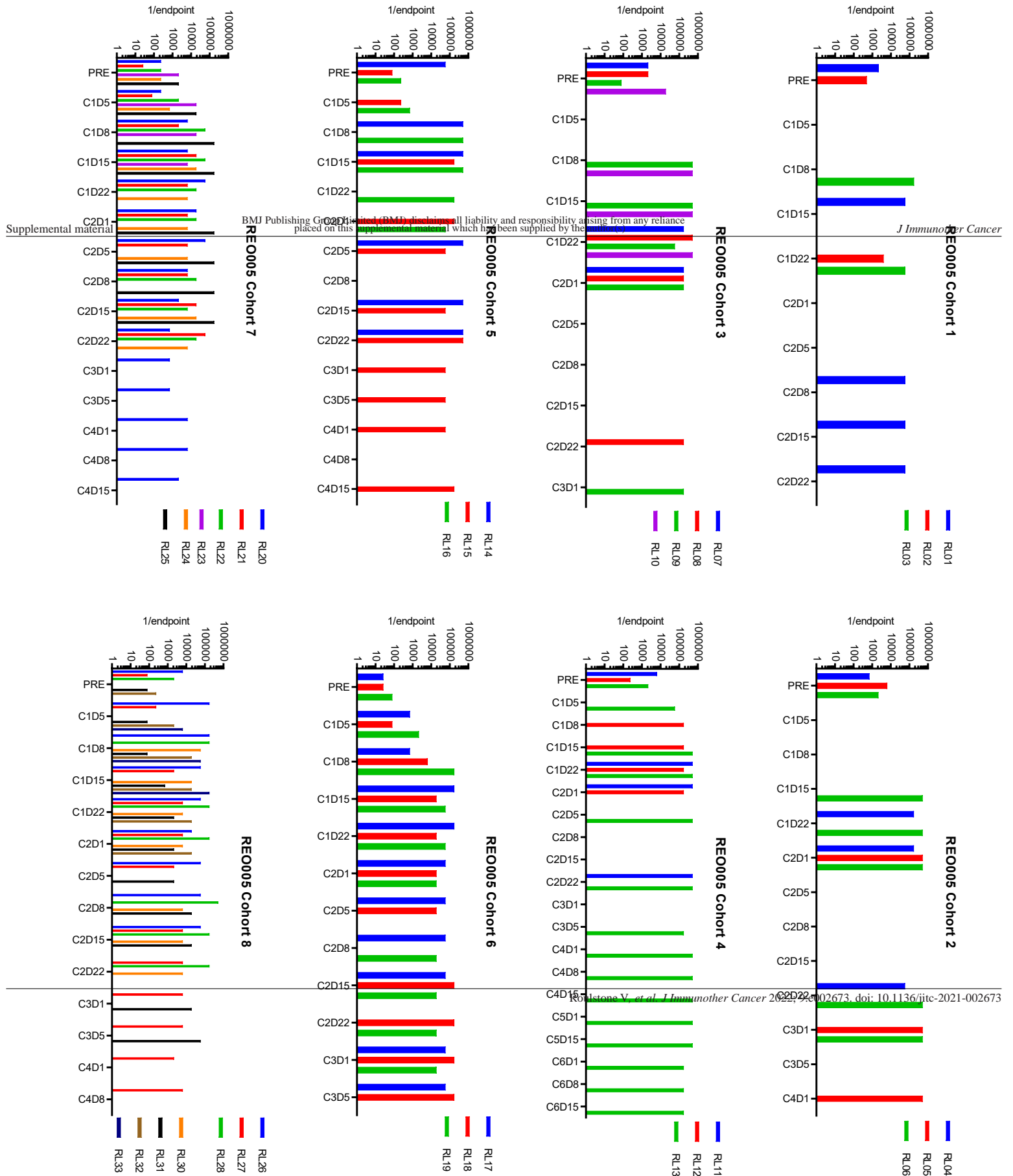

Supplement: Supplementary data [file jitc-2021-002673supp001.pdf]

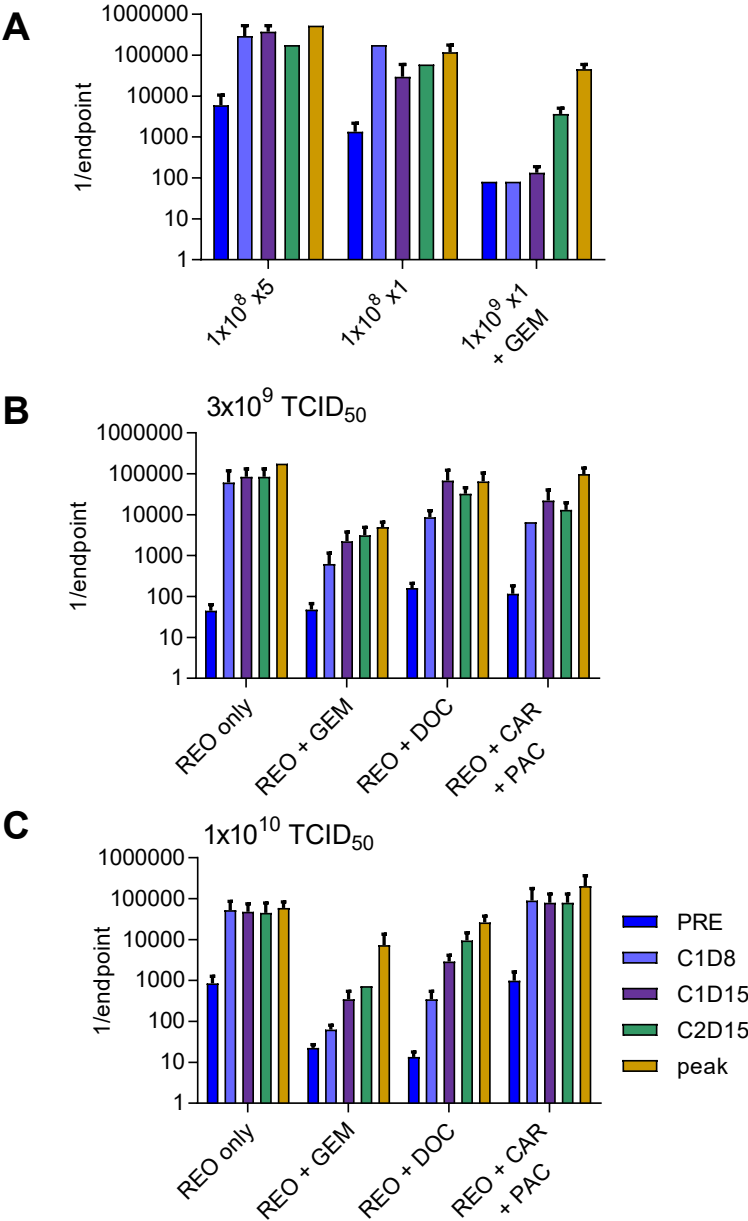

Supplement: Supplementary data [file jitc-2021-002673supp005.pdf]

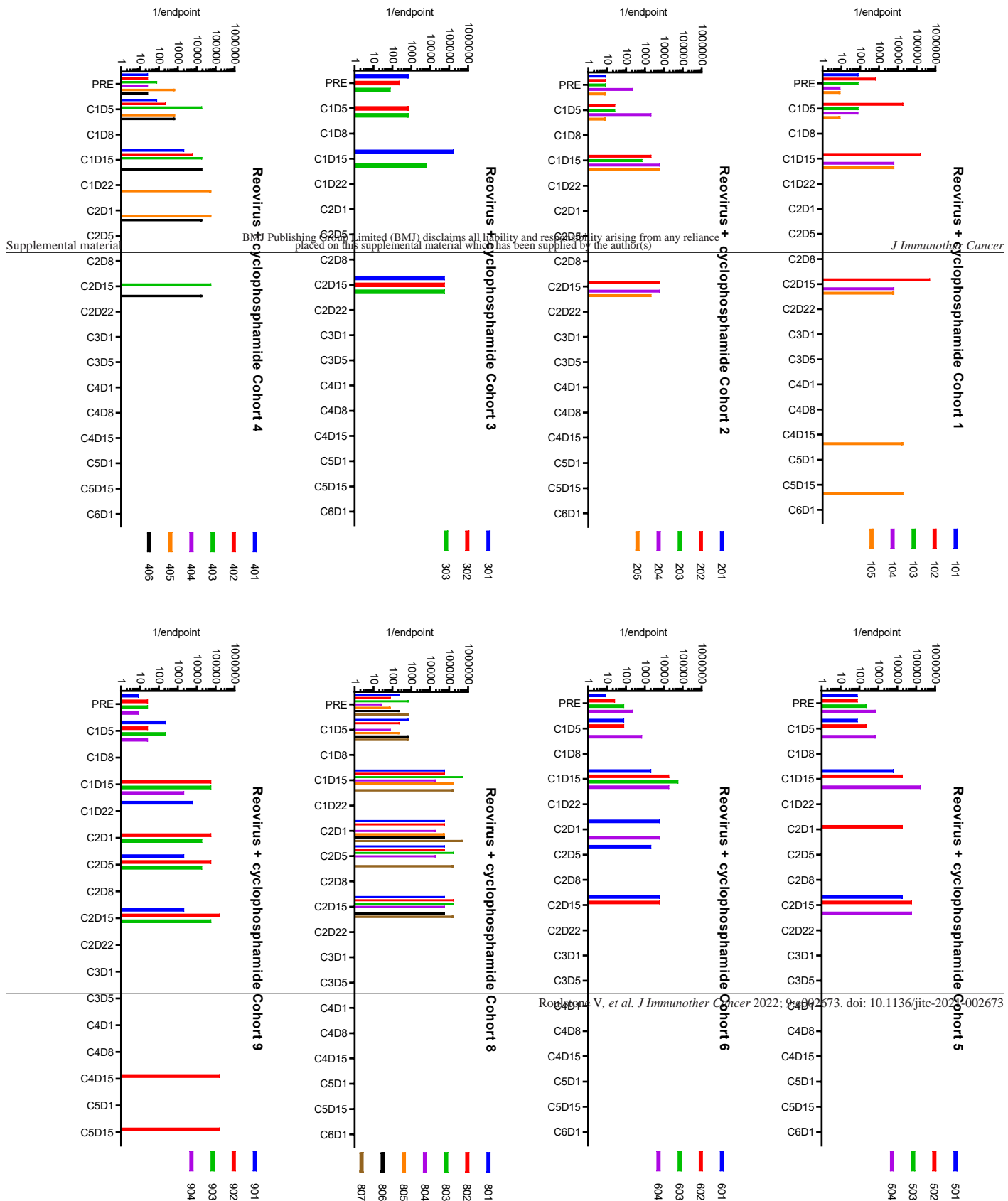

Supplement: Supplementary data [file jitc-2021-002673supp003.pdf]

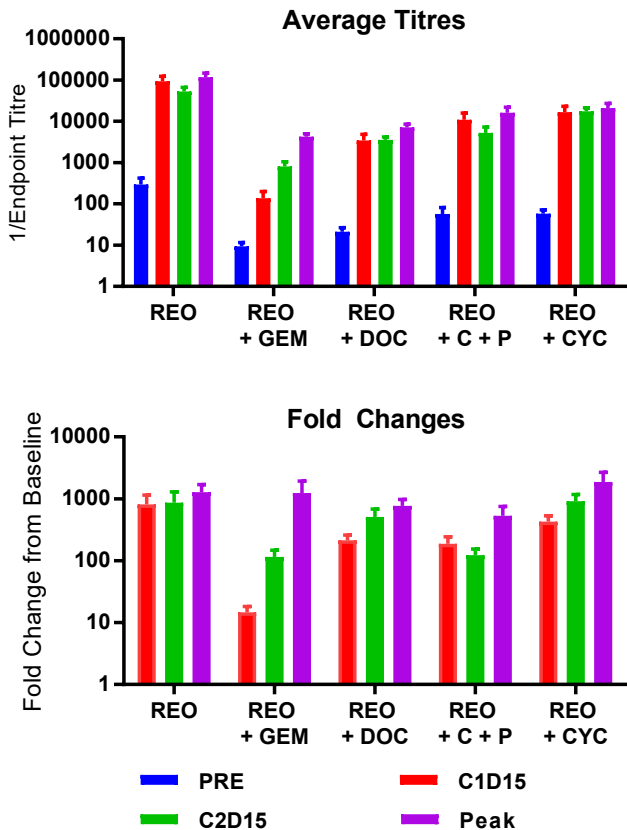

Supplement: Supplementary data [file jitc-2021-002673supp004.pdf]
