# Supplementary material for: Antiviral antibody responses to systemic administration of an oncolytic RNA virus: the impact of standard concomitant anticancer chemotherapies
Source: J Immunother Cancer. 2021 Jul 21;9(7):e002673. doi: 10.1136/jitc-2021-002673 (PMC8728387; doi:10.1136/jitc-2021-002673)
Supplement: Supplementary data [file jitc-2021-002673supp002.pdf]

BMJ Publishing Group Limited (BMJ) disclaims all liability and responsibility arising from any reliance placed on this supplemental material which has been supplied by the author(s)

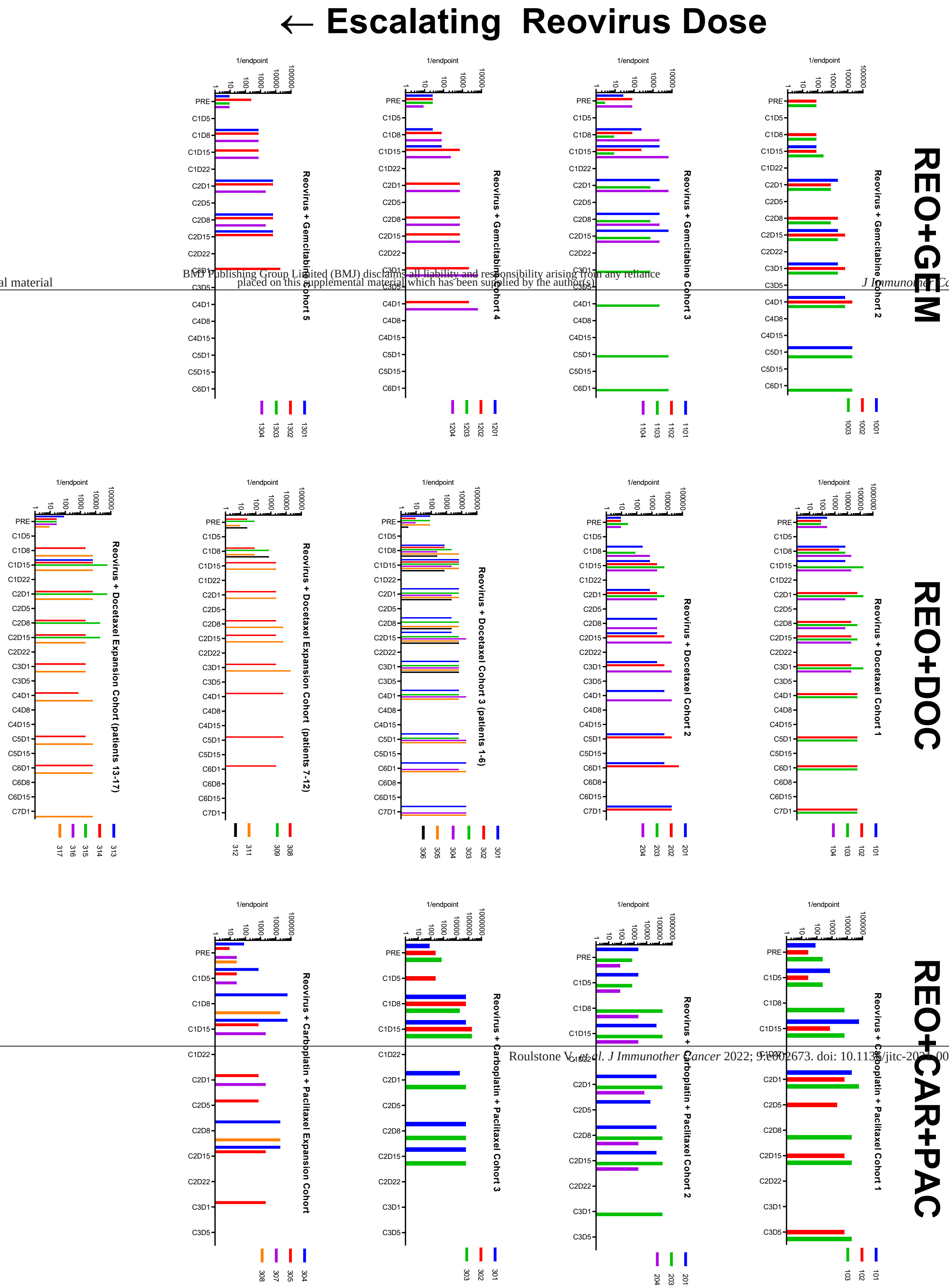

Roulstone V, et al. J Immunother Cancer 2022; 9:e002673. doi: 10.1136/jitc-2021-002673
